# Mechanisms of multi-organ damage in Wilson disease from the gut-liver-brain axis perspective: copper metabolism, gut microbiota, and metabolite communication

**DOI:** 10.3389/fmicb.2026.1868164

**Published:** 2026-06-23

**Authors:** Xue-Qiao Wang, Yan-Xin Wang, Zhao-Hao Ye, Zhi-Lin Zhang, Si-Fan Cen

**Affiliations:** Department of Neurology, The First Affiliated Hospital of Anhui University of Chinese Medicine, Hefei, Anhui, China

**Keywords:** copper toxicity, gut microbiota, microbial metabolites, neuroinflammation, Wilson disease

## Abstract

Wilson disease (WD) is an autosomal recessive disorder of copper metabolism caused by *ATP7B* mutations, leading to pathological copper deposition in the liver, brain, and cornea. Although the gut-liver-brain axis plays a role, direct copper accumulation in multiple organs remains the primary cause of tissue damage. Recent years have seen growing attention to the gut microbiota in WD pathogenesis. Copper imbalance remodels gut microbiota composition and function, while dysbiosis, in turn, affects copper absorption and excretion, forming a vicious cycle that exacerbates multi-organ damage. Copper-induced intestinal barrier disruption, lipopolysaccharide translocation, and systemic inflammation are key links connecting local copper accumulation to systemic injury. This review summarizes the genetic basis of WD, mechanisms of copper toxicity, gut microbiota alterations, and their roles in liver injury and neurodegeneration. It highlights microbiota-derived metabolites—short-chain fatty acids, tryptophan metabolites, bile acids, sulfur-containing amino acids, and branched-chain amino acids—in inter-organ communication. The bidirectional interaction between WD therapies (chelators, zinc salts, dietary interventions) and the gut microbiota is analyzed, along with microbiota-based personalized therapies. However, most current evidence derives from animal models or small cross-sectional studies; large-scale longitudinal human data are critically lacking. A deeper understanding of the gut-liver-brain axis in WD may reveal novel biomarkers and therapeutic targets.

## 1 Introduction

Wilson disease (WD) is a rare autosomal recessive disorder with a global prevalence of approximately 1:30,000. It is caused by mutations in the *ATP7B* gene, which encodes a P-type ATPase responsible for transporting copper into the Golgi apparatus for the synthesis of functional ceruloplasmin and facilitating its excretion into bile during copper overload (Roberts and Schilsky, 2023). *ATP7B* dysfunction leads to pathological copper deposition in tissues such as the liver, basal ganglia, and cornea, resulting in a spectrum of clinical manifestations, including progressive liver injury, neuropsychiatric symptoms, and the characteristic Kayser–Fleischer ring (Roberts and Schilsky, 2023). It is essential to emphasize that direct copper accumulation in these organs is the primary driver of tissue damage in WD. Over the past decades, researchers have gradually recognized that WD is not merely a simple “copper poisoning” disease but involves complex multi-organ interactions. Among these, the gut microbiota plays a critical role in copper metabolism and systemic inflammation regulation (Cai et al., 2024). This concept, known as the “gut-liver-brain axis,” provides a novel perspective for understanding the pathophysiology of WD and may reveal new therapeutic targets (Nakhal et al., 2024). This review systematically elucidates the pathogenesis of WD, with a logical thread extending from the copper metabolism disorder caused by gene mutations to gut microbiota dysbiosis, barrier dysfunction, and abnormal metabolite signaling. We further discuss emerging concepts including cuproptosis, ferroptosis, and epigenetic regulation linking diet, gut microbiota, and disease phenotype.

## 2 Genetic and pathophysiological basis of WD

### 2.1 The origin of WD: *ATP7B* mutation and copper toxicity

The fundamental cause of WD lies in mutations of the *ATP7B* gene. Over 900 disease-causing mutations have been reported, with *H1069Q* (common in European populations) and *R778L* (common in Asian populations) being the most typical (Zhou et al., 2022). These mutations lead to misfolding or loss of function of the ATP7B protein, ultimately resulting in pathological copper deposition in tissues such as the liver, basal ganglia, and cornea (Mawandji et al., 2025). Multiple studies have confirmed that this multi-organ copper accumulation is the principal cause of tissue injury, particularly in the liver, kidney, and brain (Alghamdi et al., 2026; Dang et al., 2024; Jing et al., 2020). Copper toxicity is not mediated through a single pathway but damages cells through multiple synergistic mechanisms.

At the subcellular level, mitochondrial damage from copper accumulation is a core mechanism of its toxicity. Within cells, copper is mainly distributed in the cytoplasm and organelles, with mitochondria being a key target (Borchard et al., 2018). Mitochondrial autophagy, biogenesis, fusion, and fission collectively determine mitochondrial quantity. Copper accumulation inhibits respiratory chain complex activity and reduces the expression of the mitochondrial fusion proteins Mfn1 and Mfn2, thereby inducing mitochondrial damage (Wang et al., 2024b). The specific molecular mechanism of this process involves copper ions interfering with the mitochondrial fission/fusion dynamic balance: excess copper can directly bind to specific proteins on the mitochondrial membrane, altering their conformation, subsequently activating Drp1-mediated excessive mitochondrial fission while inhibiting Mfn1/2-mediated fusion, ultimately leading to mitochondrial network fragmentation and dysfunction (Li et al., 2020). Another *in vitro* study also confirmed that mitochondrial copper accumulation in cardiomyocytes induces mitochondrial damage, leading to elevated Cyt-C levels and inducing cardiomyocyte apoptosis (Pan et al., 2022). Mitochondrial damage not only causes energy metabolism disorders but also releases substantial reactive oxygen species (ROS), forming a vicious cycle of oxidative stress. Copper also induces cytosolic escape of mitochondrial DNA and activation of the cGAS-STING-NLRP3 pathway-dependent pyroptosis in C8-D1A cells (Shi et al., 2024).

Similarly, copper overload significantly elevates oxidative stress markers such as ROS and malondialdehyde and promotes cell death by upregulating apoptotic pathways like Bax and Caspase-3 (Chen, K. et al., 2023). This process is closely linked to mitochondrial damage: the damaged mitochondrial electron transport chain is the primary ROS source, and ROS further attacks mitochondrial membrane lipids and DNA, exacerbating mitochondrial dysfunction (Zhang et al., 2019). Furthermore, the recently discovered “cuproptosis” pathway reveals another unique mechanism of copper toxicity: copper directly binds to lipoylated proteins in the tricarboxylic acid cycle, inducing their aggregation and loss of function, thereby triggering proteotoxic stress and cell death (Kim et al., 2011). Given the central role of copper accumulation in WD, the recently defined cuproptosis pathway likely contributes significantly to hepatocyte and neuronal loss (Teschke and Eickhoff, 2024). Cuproptosis is triggered by direct binding of copper ions to lipoylated mitochondrial enzymes, particularly dihydrolipoamide S-acetyltransferase (DLAT), a key component of the pyruvate dehydrogenase complex (Ma et al., 2024). Copper induces aggregation of DLAT and subsequent loss of iron–sulfur cluster proteins, leading to proteotoxic stress and ultimately cell death, independent of apoptosis or necroptosis (Brancaccio et al., 2017). In WD, excessive mitochondrial copper accumulation has been documented in hepatocytes and basal ganglia neurons, providing a permissive environment for cuproptosis (Zischka and Einer, 2018). Moreover, copper overload has been shown to downregulate the copper exporter ATP7B and upregulate the importer SLC31A1 (CTR1), thereby disrupting copper homeostasis and further sensitizing cells to cuproptotic stimuli (He et al., 2025). Notably, the dependence of cuproptosis on mitochondrial metabolism aligns with the high energy demand of the liver and brain—the two most affected organs in WD (Jiang et al., 2026). Thus, pharmacological targeting of cuproptosis (e.g., using copper chelators with mitochondrial accessibility or inhibitors of DLAT aggregation) may offer a novel therapeutic avenue beyond conventional systemic chelation.

In addition to copper, disruption of iron metabolism has been consistently observed in WD (Gromadzka et al., 2021; Pfeiffenberger et al., 2012). This iron dyshomeostasis—manifesting as increased hepatic and brain iron content, altered ferritin levels, and abnormal transferrin saturation—may contribute to ferroptosis, an iron-dependent regulated cell death distinct from apoptosis and cuproptosis (Teschke and Eickhoff, 2024). Ferroptosis is primarily driven by iron-catalyzed lipid peroxidation of polyunsaturated fatty acids (PUFAs) in cellular membranes, leading to membrane rupture (Ma et al., 2022). The key executioner of ferroptosis is glutathione peroxidase 4 (GPX4), which reduces lipid hydroperoxides using glutathione (GSH; Chen, 2026). In WD, copper overload has been shown to deplete GSH via direct binding and enhanced consumption under oxidative stress, thereby impairing GPX4 activity (Wang et al., 2023a). Furthermore, copper can inhibit the cystine/glutamate antiporter system Xc^−^, limiting cysteine availability for GSH synthesis (Kamiya et al., 2025)—a shared vulnerability with ferroptosis. Beyond GSH depletion, copper promotes the accumulation of lipid peroxidation substrates by upregulating ACSL4 (acyl-CoA synthetase long-chain family member 4) (Zhao et al., 2026), an enzyme that enriches membranes with PUFAs and sensitizes cells to ferroptotic triggers (Kuwata and Hara, 2019). In human non-alcoholic fatty liver disease (NAFLD) patients, variants of the ceruloplasmin (CP) gene have been shown to be independently associated with hyperferritinemia, increased hepatic iron storage, and more severe liver fibrosis, suggesting that genetic determinants of copper-iron crosstalk can influence clinical outcomes (Corradini et al., 2021). Collectively, the above evidence suggests that copper overload may sensitize cells to ferroptosis by depleting GSH, impairing GPX4 activity, and upregulating ACSL4, while concurrent iron dyshomeostasis further promotes lipid peroxidation. The potential interplay between cuproptosis and ferroptosis—where copper directly drives one death pathway while indirectly sensitizing cells to another—represents a critical area for future investigation (Gromadzka et al., 2024). Dual inhibition of both pathways may yield synergistic therapeutic benefits.

In the nervous system, copper can cross the blood–brain barrier (BBB) into the brain, upregulating GFAP and Caspase-3, leading to inflammation and apoptosis of striatal cells through oxidative stress and excitotoxicity (Kalita et al., 2020). Additionally, copper can permeate the BBB and move within the brain, causing elevated oxidative stress, *α*-synuclein aggregation, and lipid peroxidation via ROS-dependent pathways, subsequently leading to nerve injury (Behl et al., 2022). Notably, copper deposition in the brain is not uniformly distributed; it has high affinity for the basal ganglia (especially the putamen and globus pallidus), where copper-induced neurotoxicity—including mitochondrial damage, oxidative stress, demyelination, and iron-laden macrophage infiltration—directly contributes to the typical movement disorders in WD patients (Dusek et al., 2019).

Although copper is primarily excreted via bile, the portion that enters the bloodstream affects other organs. Chronic copper deposition disrupts the secretory function of perivascular adipose tissue in the mesentery, increasing the secretion of TNF-*α* and PAI-1 in the mesentery and elevating serum angiotensin II levels (Mawandji et al., 2025). Copper increases ROS levels in BV2 cells, activates the NF-κB classical inflammatory pathway, and increases the release of inflammatory mediators such as NLRP3 and caspase-1 (Zhou, Q. et al., 2022). This is particularly significant in patients with diabetes and hyperlipidemia (Zhao et al., 2025). Increased liver copper content accelerates physiological free radical reactions and further leads to oxidative stress (Musacco-Sebio et al., 2014). Persistent oxidative stress in hepatocytes accelerates apoptosis and necrosis by damaging lipids, proteins, and DNA (Yang et al., 2016). These modifications ultimately lead to liver dysfunction (Cichoż-Lach and Michalak, 2014). Notably, copper ions can stimulate Caco-2 intestinal cells to produce IL-8, reducing barrier integrity, thereby increasing the apparent permeability coefficient and copper translocation (Ude et al., 2017). Moreover, copper catalyzes the oxidation of the Aβ peptide, inducing oxidative stress and subsequent damage to brain nerve cells (Tomasello et al., 2025).

Together, these findings indicate that tissue damage in WD is a systemic outcome of multiple pathways, including mitochondrial dysfunction, oxidative stress, apoptosis, cuproptosis, ferroptosis, and inflammation. The triggering and amplification of this systemic damage are also related to gut microbiota imbalance.

## 3 Gut microbiota: the overlooked regulator of copper metabolism

In WD research, the gut microbiota has long been neglected. However, copper itself possesses broad-spectrum antimicrobial activity and is a natural regulator of gut microbiota composition. Studies have found that dietary copper can significantly alter the intestinal mucosal barrier function and microbial abundance, particularly the proportion of Firmicutes (Song et al., 2018), which are crucial for degrading dietary fiber and maintaining host health (Sun et al., 2023). This finding suggests that copper intake is itself an important environmental factor shaping the gut microbiota, and its alteration may precede the clinical symptoms of WD (Gu et al., 2024).

In WD patients, mutations in the *ATP7B* gene lead to impaired ceruloplasmin synthesis and biliary copper secretion, resulting in the accumulation of substantial non-ceruloplasmin-bound “free copper” in the blood. This hypercupremic state can indirectly affect the gut microbial community by altering the intestinal microenvironment or enteric substances (Dastych et al., 2021). This persistent high-copper environment exerts strong selective pressure on the gut microbiota. Similarly, copper oxide nanoparticles in food have been shown to disrupt the intestinal barrier function and induce microbial dysbiosis, even leading to non-alcoholic fatty liver disease (Jiang, L. et al., 2024).

Excess copper promotes the catabolic activity of Gram-positive bacteria and the resistance of Gram-negative bacteria (Yang et al., 2022). The molecular basis for this differential effect lies in the outer membrane structure of Gram-negative bacteria, which confers greater natural resistance to heavy metal ions (Yang et al., 2022). Fecal metabolomics results have also demonstrated dose-dependent effects of copper exposure on *α* and *β* diversity, reducing the abundance of probiotics and the Firmicutes/Bacteroidetes ratio, and altering the abundance of bacteria related to lipid metabolism and intestinal inflammation (Dai et al., 2020). These studies indicate that copper-induced dysbiosis is not a random process but a directed remodeling of the community structure.

Certain *Lactobacillus* species (e.g., *Lactobacillus plantarum* CCFM8246) and *Lactococcus* species can chelate copper ions in the gut through biosorption and binding properties, promoting its excretion and reducing systemic absorption (Zhao et al., 2024; Tian et al., 2015). The cell wall components of these probiotics (e.g., peptidoglycan and teichoic acid) contain abundant carboxyl and phosphate groups, which are the main binding sites for copper ions (Hu et al., 2024).

### 3.1 Positive feedback loop of dysbiosis and copper metabolism

More importantly, the gut microbiota not only passively responds to copper exposure but can also actively regulate host copper metabolism. In the colon of antibiotic-treated mice, the expression of the copper importer CTR1 and the copper transporter ATP7A was significantly downregulated, leading to marked impairment of copper metabolism (Miller et al., 2019). This finding reveals the regulatory role of microbiota on host intestinal copper transporter expression and metal homeostasis (Dzyhovskyi et al., 2025).

The metabolic modification of bile acids by the gut microbiota also influences the bile acid profile, thereby modulating host metabolism. For example, a study based on transcriptomics and metabolomics found that hepatic ceruloplasmin deficiency significantly restores bile acid metabolism during non-alcoholic steatohepatitis by upregulating Cyp7a1 and Cyp8b1, thereby alleviating liver injury (Jiang, Q. et al., 2024). Since the enterohepatic circulation of bile acids is a significant driving force for copper excretion, microbial regulation of the bile acid pool indirectly affects copper excretion efficiency (Sarode et al., 2019). Notably, the gut microbiota’s response to copper is highly dependent on the presence of dietary fiber: high doses of copper nanoparticles reduce various microbial enzyme activities and short-chain fatty acid levels, while fiber supplementation can effectively reverse these changes (Fotschki et al., 2026). Furthermore, the gut microbiota plays a crucial role in regulating the gut vascular barrier. Compared to germ-free mice, mice colonized with normal microbiota exhibit a more developed enteric glial cell network, increased vascular density, and elevated expression of the vascular marker gene *Ang1* (Sun et al., 2021). However, certain gut bacteria can also induce coagulation activation and subsequent vascular remodeling by activating the tissue factor-PAR1 signaling loop, further disrupting gut vascular barrier homeostasis (Reinhardt et al., 2012).

Collectively, this evidence indicates that the gut microbiota is not a passive victim but an active participant in the progression of WD. The existence of this positive feedback loop implies that simply reducing systemic copper levels may not be sufficient to completely halt disease progression; concurrent intervention targeting the gut microbiota is necessary to break this vicious cycle.

## 4 Intestinal barrier dysfunction in WD

In the multi-organ damage process of WD, the integrity of the intestinal barrier is a critical node connecting local copper toxicity to the systemic inflammatory response (Fontes et al., 2024). Under normal conditions, the intestinal barrier comprises multiple synergistic layers, including the mucus layer, the intestinal epithelial barrier, and the gut vascular barrier. However, in WD, copper toxicity can damage all three layers.

### 4.1 Multi-layered structure of the intestinal barrier and general damage characteristics in WD

Under normal conditions, the outermost layer is the mucus layer secreted by goblet cells, which restricts direct contact between epithelial cells and luminal bacteria or toxins (Zhang et al., 2023). Mucus layer thickness gradually increases along the intestine, reflecting the increasing abundance of resident microbiota (Shan et al., 2013); concurrently, mucus provides nutrient sources and colonization niches for the microbiota, and disruption of this balance can lead to infection and inflammatory responses (Johansson et al., 2015).

The middle layer is the intestinal epithelial barrier, composed of tight junction proteins, the mucus layer, and immune cells. The gut microbiota regulates tight junction gene expression, maintains epithelial cell renewal, and preserves barrier integrity through the production of short-chain fatty acids, indoles, and polyphenol metabolites (Fu et al., 2025), and restricts the diffusion of harmful substances into the body (Brescia and Rescigno, 2021; Odenwald and Turner, 2017).

The innermost layer is the gut vascular barrier at the level of the intestinal vascular endothelium, the final obstacle before microorganisms or toxins enter the systemic circulation and reach distant organs. The endothelial cells of the gut vascular barrier, together with pericytes and enteric glial cells, form the gut vascular unit, reducing paracellular molecule transport via junctional complexes to maintain barrier integrity (Zhong et al., 2021). In a healthy state, these three layers work synergistically to allow selective nutrient absorption while restricting harmful substances from entering the body. However, in WD, copper toxicity can damage the multi-layered structure of the intestinal barrier and the intestinal environment through multiple interrelated molecular pathways (Gao et al., 2020; Gao et al., 2023). Indeed, one study observed significantly increased intestinal permeability in WD patients compared to healthy controls (Fontes et al., 2024).

### 4.2 Direct copper damage to the mucus layer and epithelial barrier

Specifically in the mucus layer, copper ions can directly inhibit the differentiation and maturation of goblet cells, reducing the synthesis and secretion of MUC2 mucin, leading to mucus layer thinning and weakening its protective effect on epithelial cells (Ude et al., 2021). At the epithelial barrier level, copper activates NF-κB and MAPK inflammatory signaling pathways, promoting the release of pro-inflammatory factors such as TNF-*α* and IL-1β (Liu et al., 2020). These inflammatory factors subsequently activate myosin light chain kinase (MLCK), inducing phosphorylation and endocytosis of tight junction proteins, thereby disrupting the junctional complexes between epithelial cells (Liu et al., 2020). Concurrently, copper increases paracellular permeability through cytoskeletal actin stress fiber formation and cell contraction, as well as by inhibiting P-glycoprotein (P-gp) (Liu and Chen, 2004). Additionally, copper has direct cellular toxicity, activating intracellular oxidative stress pathways and increasing ROS levels (Vo et al., 2024). This local inflammatory response directly induced by copper further destabilizes tight junction proteins and increases paracellular permeability (Fontes et al., 2024). Meanwhile, liver damage and portal hypertension resulting from hepatic copper accumulation can cause impaired intestinal venous return, intestinal wall congestion, and edema, further compromising intestinal mucosal integrity (Nicoletti et al., 2019).

### 4.3 Copper disruption of the gut vascular barrier

At the gut vascular barrier level, inhibition of the canonical Wnt/*β*-catenin signaling pathway is a critical mechanism of barrier disruption (Dong et al., 2022). Under WD pathological conditions, copper accumulation can upregulate Wnt antagonists such as Dickkopf-1 (DKK1), inhibiting β-catenin nuclear translocation. This leads to downregulation of the tight junction protein Claudin-5 and upregulation of PV-1 (a plasmalemma vesicle protein regulating basal vascular permeability), resulting in loose endothelial cell junctions and a significant increase in gut vascular barrier permeability (Shuhan et al., 2025).

### 4.4 Copper-induced mitochondrial dysfunction and energy metabolism disorders in intestinal epithelial cells

While barrier structural integrity is compromised, the direct toxic effect of copper on intestinal epithelial cells exacerbates barrier dysfunction at the subcellular level. Intestinal damage resulting from *ATP7B* dysfunction may not simply depend on elevated total tissue copper levels but rather reflects abnormal subcellular copper distribution—because *ATP7B* deficiency prevents effective pumping of copper into the Golgi or secretion into bile, leading to abnormal cytoplasmic accumulation and selective attack on specific organelles. Mitochondria are a primary target of copper toxicity (Garza et al., 2023). Copper overload leads to crosslinking and structural disruption of liver mitochondrial membranes, accompanied by impaired activity of respiratory chain complexes and disturbances in energy metabolism, ultimately resulting in hepatocyte injury and disease progression (Zischka et al., 2011). The molecular basis of this ultrastructural damage lies in excess copper ions directly binding to mitochondrial cardiolipin, inducing abnormal opening of the mitochondrial permeability transition pore (mPTP), leading to mitochondrial membrane potential collapse and ATP synthesis deficiency (Zhang et al., 2026).

Mitochondrial dysfunction often results in energy metabolism disorders. Proteomic analysis revealed significant downregulation of oxidative phosphorylation-related proteins (e.g., NDUFS1, SDHB, UQCRC2), tricarboxylic acid cycle enzymes (e.g., CS, IDH2), and key enzymes of fatty acid *β*-oxidation (e.g., ACADM, HADHA) in WD intestinal tissues (Medici et al., 2016). Since intestinal epithelial cell renewal and tight junction maintenance are highly dependent on ATP supply, energy metabolism disorders directly weaken the epithelial barrier’s repair capacity and structural stability (Chen et al., 2025). Concurrently, lipidomic analysis of clinical samples and animal models shows varying degrees of triglyceride and cholesterol metabolism abnormalities in WD (Wu et al., 2024; Huster and Lutsenko, 2007). Given that the lipid composition of the intestinal epithelial cell membrane (especially the lipid raft structure enriched in sphingolipids and cholesterol) is crucial for tight junction protein localization and function, copper-induced lipid metabolism disruption may be another important mechanism of barrier dysfunction (Sarode et al., 2023; Pierson et al., 2018). Notably, treatment with the high-affinity copper chelator methanobactin in WD rats and human WD intestinal epithelial cells restored intestinal epithelial mitochondrial function, rebuilt epithelial integrity, and alleviated intestinal inflammation (Einer et al., 2023)—a mechanism potentially involving activation of the mitophagy pathway (Kim et al., 2020).

### 4.5 Systemic inflammatory cascade following barrier disruption: LPS translocation and gut-liver-brain Axis activation

When the intestinal epithelial barrier is compromised, bacterial products normally confined to the intestinal lumen can translocate into the portal circulation. Due to its unique anatomical proximity to the gut and receipt of the entire portal blood flow, the liver acts as the primary metabolic and detoxification organ for enteric substances. Upon entering the liver, bacterial products like lipopolysaccharide (LPS) can activate Kupffer cells and hepatic stellate cells, triggering an inflammatory response via the Toll-like receptor 4 (TLR4) signaling pathway, accelerating the production of pro-inflammatory cytokines TNF-*α* and IL-1β, and promoting liver fibrosis and hepatocyte injury (Chen et al., 2021). In the liver, LPS activates hepatic stellate cells and Kupffer cells, promoting fibrosis and inflammation; in the brain, LPS can cross the BBB or activate the vagus nerve pathway, inducing microglial activation and neuroinflammation. This inflammatory cascade, initiated by intestinal barrier disruption, represents a crucial common pathway in the multi-organ damage of WD.

In summary, in WD, copper causes synergistic damage to the various layers of the intestinal barrier through multiple mechanisms, including inhibiting mucus secretion, activating inflammatory signals, disrupting tight junctions, interfering with the Wnt/*β*-catenin vascular barrier regulatory pathway, and inducing mitochondrial dysfunction and lipid metabolism disorders. The common endpoint of these molecular events is a significant increase in intestinal permeability, laying the pathological foundation for subsequent bacterial product translocation and systemic inflammation initiation.

## 5 Metabolite-mediated gut-liver-brain communication

The influence of the gut microbiota on the host is not exerted directly on distant organs but primarily through the signaling of its metabolites across organs. These metabolites, including short-chain fatty acids (SCFAs), tryptophan metabolites, bile acids, trimethylamine N-oxide (TMAO), and derivatives of sulfur-containing and branched-chain amino acids, collectively form the material basis of the gut-liver-brain axis. In WD, copper accumulation not only directly damages tissues but also profoundly remodels the profile of microbiota-derived metabolites, thereby influencing disease progression.

### 5.1 Short-chain fatty acids

SCFAs are major products of dietary fiber fermentation by the gut microbiota, with acetate, propionate, and butyrate being the most representative (Kirschner et al., 2025). These molecules serve not only as the primary energy source for colonocytes but also possess important immunomodulatory and barrier-protective functions (Benvenuti et al., 2023). Butyrate, in particular, enhances intestinal tight junctions and reduces paracellular copper transport (Lu et al., 2026).

Butyrate plays important roles in protecting the intestinal barrier, improving hepatic lipid metabolism, and providing neuroprotection. For example, butyrate enhances intestinal barrier function and suppresses intestinal inflammation in mice by interacting with G protein-coupled receptors (GPCRs) and inhibiting histone deacetylases (HDACs) (Zhang et al., 2021). Sodium butyrate regulates the secretion of GLP-1 and PYY by intestinal L cells via activation of GPR41 and GPR43, thereby affecting systemic metabolic homeostasis (Christiansen et al., 2018). A study also found that supplementing high-fat diet-induced non-alcoholic fatty liver disease mice with *Bifidobacterium longum* BL19 increased butyrate content, CYP7A1 activity, and bile acid synthesis, while reducing inflammatory factor expression and lipid accumulation (Zhang et al., 2024). Regarding brain protection, sodium butyrate attenuated neuronal apoptosis after stroke in rats via the GPR41/PI3K/Akt pathway (Zhou et al., 2021). This indicates that butyrate exerts protective effects in the gut, liver, and brain through multiple molecular mechanisms.

Acetate can activate the hepatic AMPK/SIRT1/PGC-1α axis to alleviate ferroptosis in metabolic-associated fatty liver disease (Zhuge et al., 2025). In a rat model of lipopolysaccharide-induced neuroinflammation, acetate supplementation significantly reduced LPS-stimulated nitric oxide production and intracellular ROS levels (Moriyama et al., 2021). Similarly, acetate has protective effects in the intestinal microenvironment. Sodium acetate supplementation significantly reduced serum IL-1β, TNF-*α*, and MDA levels in LPS-induced mice and regulated intestinal inflammatory responses via the NLRP3/Caspase-1 signaling pathway (Chen et al., 2022).

Propionate also exhibits multi-organ protective effects. Propionate increased in mice via fecal microbiota transplantation can reduce lipid accumulation in the liver and adipose tissue, as well as glucose intolerance (Cao et al., 2025). Similarly, propionate can inhibit ischemia–reperfusion-induced hepatocyte inflammation and apoptosis via TLR-4 and HMGB-1 (Kawasoe et al., 2022). Validated both *in vitro* and *in vivo*, propionate strongly promotes LPS-induced IEC-6 cell migration, inhibits NLRP3 inflammasome activation, maintains intestinal barrier function, and reduces LPS-induced intestinal inflammation in rats by suppressing the TLR4/NF-κB pathway (Yang et al., 2020). Propionate plays a unique role in BBB protection. Propionate inhibits pathways associated with non-specific microbial infection via a CD14-dependent mechanism, suppresses LRP-1 expression, and protects the BBB from oxidative stress through NRF2 signaling (Hoyles et al., 2018). Propionate (but not acetate or butyrate) can reverse antibiotic-induced increased BBB permeability in mice, and *in vitro* studies show that propionate promotes tight junction protein expression in brain endothelial cells (Chenghan et al., 2025). Thus, different SCFAs exhibit functional specificity and complementarity in protecting the gut, liver, and BBB.

### 5.2 Tryptophan metabolites

Tryptophan is primarily metabolized via the kynurenine pathway and is also a major precursor for serotonin and bacterial indole pathways. Its metabolic imbalance may initiate or exacerbate neurological diseases (Huang et al., 2023). Certain tryptophan metabolites can directly or indirectly influence the physical barriers of the brain (Salminen, 2023). For example, indole and its derivatives can promote neurogenesis in the adult brain via the aryl hydrocarbon receptor (AhR) signaling pathway (Wei et al., 2021). Studies show that systemic administration or mono-colonization with indole-producing *E. coli* increases neuronal growth and neural circuit development in the mouse hippocampus, a process closely linked to AhR-mediated signaling and upregulation of *β*-catenin, Neurog2, and VEGF-alpha (Wei et al., 2021). The tryptophan-derived gut bacterial metabolite indole-3-propionic acid (IPA) increases the expression of tight junction proteins claudin-1, occludin, and ZO-1, reduces paracellular permeability, enhances the mucus barrier by increasing mucins MUC2, MUC4, and goblet cell secretory products TFF3 and RELMβ, and attenuates LPS-induced inflammatory factor expression (Li et al., 2021). Furthermore, IPA can modulate immune cell function, thereby reducing systemic inflammation-mediated damage to the nervous system (Konopelski and Mogilnicka, 2022).

Some tryptophan metabolites possess free radical scavenging and metal ion chelating abilities, thus protecting neurons from oxidative damage. For example, 3-hydroxyanthranilic acid (3-HAA) is a redox-active metabolite that, besides being a precursor to downstream quinolinic acid, can induce heme oxygenase-1 (HO-1) expression, exerting anti-inflammatory and antioxidant effects (Krause et al., 2011). More importantly, L-tryptophan can induce copper efflux, causing copper accumulation in the periplasmic space, thereby directly inhibiting ClbP enzyme activity and reducing *E. coli* toxicity (Bayne et al., 2024). This finding suggests that tryptophan metabolism might influence bacterial pathogenicity by modulating intestinal copper distribution, but whether this applies to the pathophysiology of WD and its potential regulatory role in host copper homeostasis requires experimental validation. Additionally, studies have found that gut microbiota-related tryptophan metabolites can alleviate intestinal barrier dysfunction in glucolipid metabolism disorders (Chen, Y. et al., 2023).

### 5.3 Bile acid metabolites

The metabolic transformation of bile acids (BAs) by the gut microbiota is a key chemical link connecting the gut, liver, and central nervous system. Primary bile acids synthesized in the liver and secreted into the intestine are converted by the gut microbiota through deconjugation, dehydroxylation, and epimerization into secondary bile acids such as deoxycholic acid (DCA) and lithocholic acid (LCA), and their derivatives like isoLCA and 3-oxoLCA (Shapiro et al., 2018). These microbe-derived metabolites are not merely emulsifiers for lipid digestion but are signaling molecules that cross biological barriers via the bloodstream or neural pathways, playing central roles in regulating neuroinflammation, neurotransmitter balance, and neurodegenerative diseases (Czaj et al., 2026).

The FXR signaling pathway is a crucial hub connecting bile acid metabolism, gut microbiota, and liver inflammation (Wu et al., 2025). Copper can exacerbate liver injury via the FXR signaling pathway. Specifically, copper can reduce the abundance of *Lactobacillus*, thereby upregulating serum and hepatic levels of taurine-*β*-muricholic acid. As an antagonist of the farnesoid X receptor (FXR), taurine-β-muricholic acid inhibits FXR signaling in the liver and ileum, ultimately promoting CYP7A1 transcription and increasing total bile acid concentration, driving liver inflammation (Wu et al., 2025).

Deoxycholic acid (DCA) has a well-defined disruptive effect on the intestinal barrier. DCA leads to a reduction of indoleamine 2,3-dioxygenase 1 (IDO1) in Paneth cells of high-fat diet-fed mice, causing a paracrine deficiency of the AhR ligand kynurenine in the crypts, thereby disrupting the intestinal mucosal barrier function (Liu et al., 2022). Studies have also found that DCA induces activation of the NLRP3 inflammasome, increases the production of inflammatory cytokines, causes low-grade intestinal inflammation, and significantly suppresses secretory immunoglobulin A (sIgA) levels (Liu et al., 2018). Similarly, DCA increases the production of intracellular reactive oxygen species and, accompanied by attenuation of the ERK1/2 signaling pathway, significantly downregulates the mRNA levels of intestinal barrier-related junction proteins in Caco-2 cells (Zeng et al., 2022). More importantly, DCA mediates cognitive impairment induced by a high-fat diet in mice, and hippocampal apical bile acid transporters can reduce neuronal DCA accumulation, improving neuronal apoptosis and cognitive impairment (Liu et al., 2025b). Persistent neuroinflammation is a key pathological feature in neurodegenerative diseases such as Alzheimer’s disease (AD), Parkinson’s disease (PD), and multiple sclerosis (MS; Geng et al., 2023). Conversely, studies have shown that ursodeoxycholic acid (UDCA) and tauroursodeoxycholic acid (TUDCA) possess significant neuroprotective properties, alleviating oxidative stress and mitochondrial dysfunction (Geng et al., 2023).

Notably, several secondary bile acids, including DCA and LCA, and their derivatives, are involved in the differentiation and function of innate and adaptive immune cells (Su et al., 2023). LCA and 3-oxoLCA can activate the vitamin D receptor (VDR) in immune cells (including macrophages and T cells), inducing an anti-inflammatory phenotype and inhibiting excessive Th1 cell activation, thereby alleviating the neuroinflammatory microenvironment (Makishima et al., 2002). Further research revealed that 3-oxoLCA directly binds to the key transcription factor retinoic acid receptor-related orphan receptor-γt (RORγt) to inhibit Th17 cell differentiation, while isoalloLCA increases Treg cell differentiation by generating mitochondrial reactive oxygen species, leading to increased FOXP3 expression (Hang et al., 2019). Similarly, allo-LCA can activate the G protein-coupled bile acid receptor 1 (GPBAR1) and trans-repress RORγt, preventing macrophage M1 polarization and CD4 + cell Th17 polarization (Marchianò et al., 2025). Thus, microbial modification of bile acids produces functionally diverse and even opposing metabolites, which may play distinct regulatory roles in the inflammatory milieu of WD.

### 5.4 Trimethylamine N-oxide

Trimethylamine N-oxide (TMAO) is a metabolite produced by the gut microbiota from choline and carnitine. Its effects on the liver and nervous system are complex. On one hand, TMAO maintains gut microbiota homeostasis and protects the integrity of liver sinusoidal endothelial cells by inhibiting the expression of the endothelial β1 subunit of Na^+^/K^+^-ATPase, alleviating liver fibrosis in non-alcoholic steatohepatitis (Zhou et al., 2022). TMAO intake can reduce liver injury markers in farnesoid X receptor (*Fxr*)-deficient mice and further alter bile acid/cholesterol metabolism (Miyata et al., 2024). However, conversely, reduced serum TMAO levels can ameliorate liver injury induced by L-carnitine intake (Han et al., 2024). TMAO can also induce liver fibrosis by activating the PI3K/AKT pathway, an effect that Bie Jia Jian Pill (BJJP) can delay (Cui et al., 2025). Regarding the central nervous system, midbrain organoids treated with TMAO exhibit certain neurodegenerative phenotypes, including impaired brain-derived neurotrophic factor (BDNF) signaling, loss of dopaminergic neurons, astrocyte activation, neuromelanin accumulation, as well as pathophysiological phosphorylation of *α*-synuclein and Tau protein (Lee et al., 2022). These findings suggest that the role of TMAO is tissue-specific and condition-dependent, and its net effect in the context of WD remains to be elucidated.

### 5.5 Sulfur-containing amino acid metabolism and derivatives

Taurine and glutathione (GSH), as important derivatives of sulfur-containing amino acid metabolism, primarily protect the integrity of the gut-liver-brain axis by maintaining redox balance and detoxification functions. In the gut-liver interaction, taurine mainly combines with bile acids to form taurocholate excreted into the intestine. The gut microbiota (especially *Clostridia* species) decomposes it via bile salt hydrolase (BSH) (Yang et al., 2024). The released taurine can be reabsorbed by the host for hepatic recycling or further metabolized by the microbiota to produce hydrogen sulfide (Yang and He, 2019). Meanwhile, GSH synthesis depends on cysteine generated through the transsulfuration pathway from homocysteine (Hcy), and the gut microbiota indirectly affects the hepatic GSH pool by modulating cysteine availability (Agostini et al., 2025).

In terms of brain neuroprotection, GSH is recognized as the most important endogenous antioxidant in the central nervous system, capable of scavenging ROS and alleviating neuroinflammation. Due to poor GSH bioavailability, compounds carrying GSH have been developed as brain neuro-antioxidants (Raza et al., 2022; Chiang and Nicol, 2022). Regarding the intestinal barrier, reduced GSH and GPX4 levels affect the efficiency of lipid peroxidation clearance, mediating ferroptosis induced by intestinal ischemia–reperfusion oxidative stress (Huangfu et al., 2025; Wang et al., 2023b). Zinc-glutathione complexes can maintain plasma GSH levels, thereby reducing alcohol-induced hepatic steatosis and intestinal barrier injury (Feng et al., 2024).

Taurine binds to TLR4 and inhibits the TLR4/NF-κB pathway, ultimately reducing the pro-inflammatory cytokines TNF-*α*, IL-6, and oxidative stress. It also enhances intestinal barrier function by increasing goblet cells and upregulating tight junction protein expression (Zheng et al., 2024). Studies have also found a synergistic effect of taurine and butyrate on RIA-mediated hydrolysis of p-NPP, jointly alleviating LPS-mediated disease (Arise et al., 2022). Taurine inhibits Fpr2-regulated macrophage M1 polarization and related pro-inflammatory cytokine production by suppressing NF-κB p65 phosphorylation (Xie et al., 2025). Taurine treatment can also attenuate autophagy in hepatic stellate cells by reducing autophagosome formation, downregulating LC3B and Beclin1 protein expression, and upregulating p62 protein expression, thereby reducing liver fibrosis (Li et al., 2024). Research has found that the histone methyltransferase NSD2 prevents intestinal barrier disruption by maintaining taurine biosynthesis, and exogenous taurine supplementation can significantly alleviate inflammatory bowel disease symptoms caused by NSD2 deficiency (Xu et al., 2024).

Homocysteine (Hcy), a key intermediate in sulfur-containing amino acid metabolism, is a central node connecting the gut microbiota, hepatic metabolism, and neurological function. In the gut-liver axis, the gut microbiota (e.g., *Faecalibaculum* and *Dubosiella*) directly participates in Hcy biosynthesis via *cbl* and *cgs* genes, while the liver is the primary site for Hcy transmethylation and transsulfuration, a process highly dependent on B vitamins synthesized by the gut microbiota as cofactors (Li et al., 2023). Elevated Hcy is not only an independent risk factor for vascular endothelial dysfunction but also activates the NLRP3 inflammasome in macrophages, promoting the release of pro-inflammatory cytokines such as IL-1β and IL-18, triggering systemic low-grade inflammation. This “gut-derived inflammatory signal” can cross the BBB via the bloodstream, exacerbating neuroinflammation and cognitive impairment (Wang et al., 2017). Clinical evidence shows that in patients with Alzheimer’s disease, Parkinson’s disease, and major depressive disorder, plasma Hcy levels are significantly negatively correlated with gut microbiota diversity and are closely linked to changes in the abundance of genera such as *Alistipes* and *Ruminococcaceae* (Rosario et al., 2021; Xu et al., 2025). Therefore, Hcy may represent a crucial bridge molecule connecting WD-associated dysbiosis with neuropsychiatric symptoms.

### 5.6 Branched-chain amino acids and aromatic amino acids

Branched-chain amino acids (BCAAs, e.g., leucine, isoleucine, valine) and aromatic amino acids (AAAs, e.g., phenylalanine, tyrosine, tryptophan), as essential amino acids, play critical roles in the physiological and pathological processes of the gut-liver-brain axis. The gut is the starting point for BCAA metabolism. Studies show that gut microbiota such as *Prevotella*, *Faecalibacterium*, and *Roseburia* directly participate in BCAA biosynthesis by carrying genes like *ilvB*, *ilvC*, and *ilvD* (Zhang, Y. et al., 2022). Research also indicates that in pathological states like Parkinson’s disease, this microbial ecology becomes dysregulated, characterized by reduced abundance of BCAA-producing bacteria and an increase in pathobionts (Zhang, Y. et al., 2022). This alteration in microbial community structure not only reduces systemic BCAA supply but also causes an imbalance in intestinal amino acid metabolism due to the breakdown of cross-feeding mechanisms (Hernández-Valles et al., 2026).

BCAAs and AAAs competitively share the large neutral amino acid transporter 1 (LAT1) for crossing the BBB. LAT1 is responsible for transporting tryptophan (a precursor of 5-HT) and tyrosine (a precursor of DA) into the brain (Zaragozá, 2020). When peripheral BCAA concentrations are elevated, they competitively inhibit the binding of AAAs to LAT1, thereby reducing their entry into the central nervous system (Fernstrom, 2005). Excessive peripheral BCAA intake can overactivate NMDA receptors, causing oxidative damage, altered antioxidant enzyme activity, and increased pro-inflammatory cytokines, leading to memory changes (Lemos et al., 2024). Similarly, BCAA accumulation can exacerbate microglia-induced neuroinflammation by activating the AKT/STAT3/NF-κB signaling pathway (Shen et al., 2023).

Research has found that tissue hypoxia and inflammatory microenvironments inhibit the activity of branched-chain *α*-keto acid dehydrogenase (BCKD), thereby hindering the conversion of BCAAs to branched-chain fatty acids (BCFAs) (Wallace et al., 2018). This results in the retention of large amounts of BCAAs in the blood, which can interfere with insulin signaling and cause fatty liver by activating the mTORC1 pathway in muscles and the liver (Ye et al., 2020).

On one hand, the gut microbiota releases BCAAs and AAAs by breaking down dietary proteins. Microbiota from phyla such as Bacteroidetes, Firmicutes, and Proteobacteria participate in the deamination and decarboxylation of phenylalanine, producing intermediates like phenylacetic acid (PAA; Dodd et al., 2017; Zhu et al., 2023). On the other hand, BCAAs can affect intestinal barrier function by modulating the expression of tight junction proteins, while abnormal AAA metabolism (e.g., phenylalanine accumulation) may induce intestinal mucosal inflammation, disrupting the material exchange homeostasis of the gut-liver axis (Chen et al., 2019).

The liver is a central hub for amino acid metabolism, regulating the systemic distribution of BCAAs and AAAs via the urea cycle and transamination reactions. Notably, when liver function is impaired, reduced urea synthesis capacity leads to elevated blood ammonia. Simultaneously, phenylalanine is converted by the gut microbiota to PAA, which is then conjugated with glutamine in the liver to form phenylacetylglutamine (PAGln). This gut-derived metabolite can enter the systemic circulation via the portal vein, and its levels are positively correlated with the severity of hepatic encephalopathy (Stravitz et al., 2018). Animal studies show that PAGln can cross the BBB and enhance platelet activation by activating adrenergic receptors (α2A, α2B, β2 AR), thereby increasing the risk of cerebrovascular thrombosis. Another study also confirmed that PAGln is highly correlated with increased platelet reactivity and thrombosis risk (Liu et al., 2025a).

At the brain axis level, an imbalance in the BCAA/AAA ratio directly affects neurotransmitter synthesis and neuroinflammation processes. Under physiological conditions, BCAAs competitively inhibit AAA transport via LAT1, reducing phenylalanine entry into the brain and maintaining normal dopamine and serotonin synthesis. In Alzheimer’s and Parkinson’s disease patients, the BCAA/AAA ratio is significantly decreased, leading to phenylalanine accumulation in the cerebrospinal fluid and brain tissue. Its metabolite, PAGln, not only promotes microglial overactivation via the TLR4/AKT/mTOR pathway but also induces abnormal phosphorylation of *α*-synuclein, accelerating neurodegeneration (Rosario et al., 2021; Liu et al., 2021). Furthermore, PAGln, as a novel signaling molecule of the gut-brain axis, shows levels significantly correlated with hippocampal phospholipid metabolism disorders in patients with mild cognitive impairment (MCI), suggesting it may participate in AD pathology by interfering with lipid metabolism (Zhang, Q. et al., 2022). Given that WD patients often exhibit liver damage and neuropsychiatric symptoms, BCAA/AAA imbalance and PAGln accumulation may be important metabolic bridges connecting liver and brain pathology.

## 6 Bidirectional interaction between WD pharmacotherapy and the gut microbiota

A complex bidirectional interaction exists between pharmacotherapy for WD and the gut microbiota, a relationship with profound implications for treatment efficacy and disease progression. The most direct evidence comes from antibiotic use. Although large-scale epidemiological data on antibiotic usage rates in WD patients are lacking, the potential disturbance of the gut microbiota by antibiotics is particularly concerning in this patient population. Antibiotics can rapidly reshape the structure of the gut microbiota within days, and restoration of certain bacterial species can take weeks to months even after cessation (Kappel et al., 2020). For WD patients, who already exhibit reduced microbial diversity and fewer SCFA-producing bacteria (Cai et al., 2024), the additional impact of antibiotics could push their gut microbiota into an even more difficult-to-reverse dysbiotic state. More importantly, antibiotic-induced reduction in microbial diversity is closely associated with impaired intestinal barrier function (Feng et al., 2019), potentially further weakening the gut barrier, promoting lipopolysaccharide translocation, and systemic inflammation. Therefore, in WD patients, antibiotic use should be strictly indicated, avoiding unnecessary exposure and considering adjunctive probiotics or microbiota-directed interventions when necessary to mitigate negative effects.

D-Penicillamine (DPA), a first-line chelator for WD, classically acts by chelating copper in the blood and promoting its urinary excretion (Gupta et al., 2024). However, a recent human PET/CT study significantly revised this understanding. Kirk et al. (2024) used oral ^64^Cu tracer PET/CT in healthy volunteers before and after DPA treatment to quantify the drug’s effect on intestinal copper absorption. Results showed that DPA treatment only slightly reduced hepatic (Fu et al., 2025) Cu uptake (approximately 25% after 15 h), indicating that DPA’s inhibitory effect on intestinal copper absorption is very limited, and its primary copper-excreting pathway remains enhanced urinary excretion. Nevertheless, the interaction of DPA with the gut microbiota extends beyond this. First, DPA itself possesses broad-spectrum antibacterial activity, and long-term use can inhibit the growth of various beneficial bacteria, including *Lactobacillus* and *Bifidobacterium*, exacerbating the dysbiosis already present in WD patients (Cai et al., 2024). Second, the gut microbiota significantly influences DPA’s therapeutic efficacy. Huang et al. (2025) found in WD model mice that although DPA treatment partially restored copper load-induced gut dysbiosis, the abundance of specific bacterial genera was strongly correlated with copper excretion efficacy. Notably, *Akkermansia muciniphila* (Akk) abundance showed a strong negative correlation with liver copper concentration. When Akk was supplemented alongside DPA treatment, liver and brain copper levels in mice further decreased, and liver function (ALT, AST) and histopathological injury improved more significantly. The potential mechanism of this synergistic effect involves Akk and other beneficial bacteria enhancing intestinal barrier function, reducing endotoxin translocation, and systemic inflammation (Grander et al., 2020), thereby creating a more favorable host environment for DPA’s copper-chelating action.

While less studied, the impact of the chelator trientine (TRI) on the gut microbiota may differ from DPA, as TRI primarily binds copper in the intestinal lumen to form an unabsorbed complex, potentially leading to a different pattern of direct effects on the microbiota compared to the systemic antibacterial activity of DPA. Future studies are needed to elucidate the specific effects of TRI on the gut microbiota composition and function in WD patients and to assess whether it can also enhance efficacy by modulating specific beneficial bacteria, similar to DPA.

Zinc salts, another first-line treatment for WD, have a mechanism of action distinct from chelators, primarily inducing the expression of metallothionein (MT) in intestinal epithelial cells and hepatocytes. MT is a cysteine-rich, low-molecular-weight protein with high affinity for copper. It binds dietary copper within enterocytes, which is then excreted upon normal shedding of these cells, thus blocking copper entry into the systemic circulation (Munk et al., 2022; Meacham et al., 2018). Sturniolo et al. (1999) confirmed in duodenal biopsies of WD patients that long-term oral zinc therapy increased intestinal MT protein concentration approximately 15-fold, and MT levels were highly positively correlated with intestinal zinc concentration, providing direct human evidence for the mechanism of zinc-mediated copper absorption blockade. The effect of zinc salts on the gut microbiota is also noteworthy. On one hand, MT induction is a protective response to zinc exposure, and the gut microbiota may adapt to a high-zinc environment through similar mechanisms. On the other hand, zinc is an essential trace element for the growth of many bacteria; both deficiency and excess can lead to dysbiosis. Zinc supplementation may indirectly promote the growth of SCFA-producing bacteria (e.g., *Faecalibacterium prausnitzii*) and enhance intestinal barrier function by correcting potential zinc metabolism abnormalities in WD patients (Wang et al., 2024a). Importantly, zinc gluconate can reverse antibiotic- and LPS-induced intestinal barrier injury (Wang et al., 2024a).

Recently, novel therapeutic strategies targeting the gut-liver-brain axis have been explored. Ammonium tetrathiomolybdate (TTM) forms a copper-molybdenum-protein complex in the intestine, preventing copper absorption, and has shown potential in WD patients with neurological manifestations (Posada and Ramos, 2022). Theoretically, such drugs targeting intestinal copper absorption may have a smaller impact on the systemic microbiota compared to systemic chelators. Similarly, the liver-targeted chelator WTX101 may preserve more gut microbiota homeostasis due to its hepatic selectivity (Weiss et al., 2018). However, systematic data on the specific effects of these novel drugs on the gut microbiota are still lacking.

Notably, WD patients often require multiple medications due to liver dysfunction, neuropsychiatric symptoms, or comorbidities, including proton pump inhibitors, antidepressants or antipsychotics, and potentially Chinese patent medicines for liver protection. The interactions of these drugs with the gut microbiota should not be overlooked (Tian et al., 2023). PPIs can promote the intestinal colonization of oropharyngeal bacteria and inhibit commensal bacteria (Xiao et al., 2024), a phenomenon that may also occur in WD patients and potentially worsen dysbiosis. Furthermore, dietary interventions (e.g., low-copper diet, high-fiber diet), as important components of WD management, are among the strongest environmental factors influencing the gut microbiota (Gong et al., 2023). High-fiber diets promote the growth of SCFA-producing bacteria and improve intestinal barrier function (Ballout et al., 2025), while low-copper diets might indirectly alter the sources of dietary fiber and prebiotics by restricting specific foods (e.g., shellfish, nuts) (Creedon et al., 2022; Kelly Souza Silveira et al., 2024).

Overall, research on the bidirectional interaction between WD drugs and the gut microbiota is still in its early stages. Most evidence comes from animal models or small-scale cross-sectional studies, lacking large-scale, multi-center, multi-omics cohort studies. Future longitudinal studies should simultaneously collect fecal, blood, and clinical data, combined with metagenomic and metabolomic analyses, to disentangle the relative contributions of the disease itself, drugs, and diet to the microbiota. Only upon a thorough understanding of these complex interactions can chelator and zinc salt selection and dosing be optimized based on microbiota profiles, potentially even using adjunctive probiotics or fecal microbiota transplantation.

In addition to drug-microbiota interactions, emerging evidence indicates that diet and gut microbiota can influence epigenetic processes in WD. Mordaunt et al. demonstrated that in a mouse model of Wilson disease, dietary choline supplementation alters hepatic DNA methylation patterns and affects thioredoxin system-related gene expression, thereby contributing to phenotypic changes (Dong et al., 2025). This suggests that nutritional factors acting through the gut-liver axis may have long-lasting epigenetic effects. Given that the gut microbiota is a key modulator of nutrient bioavailability and metabolism, it is plausible that microbiota-derived signals contribute to epigenetic regulation in WD (Woo and Alenghat, 2022). These findings open new possibilities for dietary and microbiota-based epigenetic interventions as adjunctive therapies in WD management.

## 7 Conclusions and future perspectives

This review systematically elucidates the cascade pathological mechanisms from a single gene mutation to multi-organ damage in WD. Current evidence indicates that WD is not merely a simple “copper poisoning” disease resulting from *ATP7B* gene mutations but rather a systemic pathological process driven by the gut-liver-brain axis. The core vicious cycle involves: copper accumulation remodels the gut microbiota, leading to dysbiosis and altered metabolite profiles; this subsequently damages the intestinal mucus, epithelial, and vascular barriers, causing LPS translocation and abnormal inter-organ communication via microbial metabolites; finally, by activating systemic inflammation and oxidative stress, it exacerbates liver injury and neurodegeneration.

Nevertheless, it is important to reiterate that direct copper accumulation in target organs remains the primary initiator of tissue damage, and the gut-liver-brain axis acts as a critical amplifier and modulator of disease progression. Furthermore, emerging concepts such as cuproptosis, ferroptosis, and epigenetic regulation by diet and gut microbiota add new layers of complexity to WD pathogenesis. These mechanisms may offer novel therapeutic targets beyond conventional copper chelation or zinc therapy.

However, several important limitations must be explicitly recognized. First, the majority of findings on gut microbiota alterations in WD are derived from animal models (primarily *Atp7b*^−^/^−^ mice) or small-scale, cross-sectional human studies with limited sample sizes. Large-scale, multi-center, longitudinal human cohort studies integrating metagenomic, metabolomic, and detailed clinical phenotyping are urgently needed to establish causal relationships. Second, confounding factors such as heterogeneous treatment regimens, dietary variations, and concurrent medications have not been adequately controlled in most studies. Third, the mechanistic understanding of microbiota-derived metabolites in WD remains largely correlative; direct interventional studies in humans are required to test causality. Therefore, while the current evidence is promising, it should be interpreted with caution.

Future research should focus on establishing large-scale, multi-center WD patient cohorts, integrating multi-omics approaches to investigate causal effects of specific microbiota metabolites. The interplay between copper, iron, and other metals, as well as the potential for microbiota-targeted epigenetic interventions, represents a particularly promising direction. Given the current reliance on animal models and small human studies, the translational potential of microbiota-based diagnostics and therapies for WD remains to be validated in large, prospective human cohorts.
